# tRUBY: A convenient *in planta* tool for the detection of protein-DNA and protein-protein interactions

**DOI:** 10.1016/j.mocell.2026.100357

**Published:** 2026-04-10

**Authors:** Sangyun Kim, Heebak Choi, Tae Gyu Yi, Sun-Hwa Ha

**Affiliations:** Graduate School of Green-Bio Science, College of Life Sciences, Kyung Hee University, Yongin 17104, Republic of Korea

**Keywords:** Betalain biosynthesis, Noninvasive plant reporter, Thosea asigna virus, Tobacco

## Abstract

Elucidating molecular interactions such as protein–DNA (PDIs) and protein–protein (PPIs) has traditionally relied on yeast-based 1-hybrid (1H) and 2-hybrid (2H) systems. To provide an alternative platform that better reflects the native cellular environment of plants, we optimized the tRUBY reporter system for 1H and 2H assays in *Nicotiana benthamiana*, enabling direct *in planta* analysis of PDIs and PPIs. Specifically, the 2A peptide sequence used for co-expressing the 3 betalain biosynthetic genes—responsible for the visible RUBY coloration—was replaced with T2A from the *Thosea asigna* virus in place of P2A or F2A from mammalian-pathogenic *Picornaviridae* viruses, improving biosafety for agricultural applications. The resulting tRUBY-1H and tRUBY-2H systems operate under near-physiological conditions with physiologically relevant expression levels, enabling quantitative, multiplexed, and directly compatible protein-level analyses, thereby offering high sensitivity and flexibility for advanced molecular studies. Ultimately, these systems demonstrate that the streamlined, cost-effective, and visually scorable *in planta* platform provided by RUBY is well-suited for intuitive, non-destructive monitoring of molecular interactions in plant tissues.

## Graphical Abstract

**Figure:**
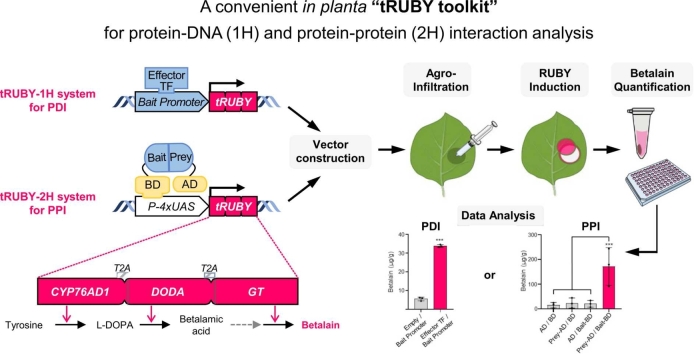

## INTRODUCTION

Protein-DNA interactions (PDIs) and protein-protein interactions (PPIs) are fundamental to cellular regulation, including transcription, signaling, and development. Various techniques have been developed for their analysis, such as yeast one-hybrid (1H), electromobility shift assay, chromatin immunoprecipitation sequencing (ChIP), and dual-luciferase assays for PDIs, as well as yeast 2-hybrid (2H), split-ubiquitin-based yeast 2H, bimolecular fluorescence complementation (BiFC), split-luciferase complementation, pull-down assay, and proximity labeling approaches such as TurboID for PPIs (Fields and Song, 1989, Fujikawa and Kato, 2007, Hellens et al., 2005, Park and Kim, 2025). However, these methods often rely on artificial systems that fail to reflect the native *in planta* environment—such as chromatin state, co-factor availability, subcellular localization, or physiological signaling—and typically require extensive cloning, stable transformation, and specialized equipment or chemical treatment. Consequently, multiple interaction analysis tools are often employed in parallel, frequently yielding conflicting results. These limitations highlight the need for *in planta* platforms that preserve native regulatory contexts while supporting both visual and quantitative analyses with minimal instrumentation, thereby offering a cost-effective alternative to conventional methods (Lalonde et al., 2008, Xing et al., 2016).

**Fig. 1:**
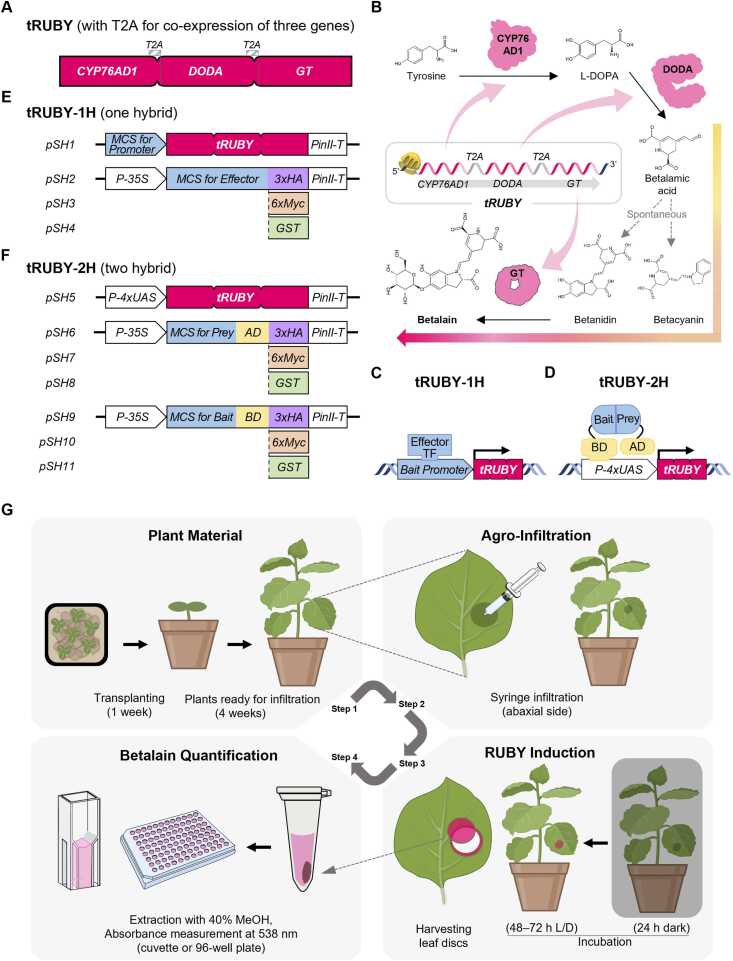
*Schematic diagrams and optimized workflow of the tRUBY reporter systems.* (A) Schematic of the tRUBY cassette utilizing T2A sequences to facilitate the stoichiometric co-expression of betalain biosynthetic genes. (B) Betalain biosynthetic pathway reconstituted by the tRUBY system. The diagram illustrates the conversion of tyrosine into betalain pigment via the T2A-linked multi-enzyme complex. (C, D) Schematic working mechanisms of the tRUBY-1H (C) and tRUBY-2H (D) systems. (E, F) Maps of the optimized binary vectors. The series includes the tRUBY-1H vectors (*pSH1-pSH4*) (E) and the tRUBY-2H vectors (*pSH5-pSH11*) (F). (G) Schematic workflow of the *Agrobacterium*-mediated transient expression assay. The procedure summarizes the entire process, including plant preparation, agro-infiltration, reporter induction under standard light/dark cycles (L/D), and betalain quantification.

The RUBY reporter system was developed as a tool for monitoring gene expression and plant transformation in *Arabidopsis* and rice, driven by five promoters, offering an output detectable by the naked eye and presenting a promising solution to several technical challenges (He et al., 2020). RUBY consists of 3 betalain biosynthetic enzymes—cytochrome P450 76AD1 (CYP76AD1), L-DOPA 4,5-dioxygenase (DODA), and a glucosyltransferase (GT)—which are co-expressed as a single transcript being linked by 2A “ribosomal skipping” peptides, such as P2A from Porcine teschovirus-1 or F2A from foot-and-mouth disease virus, both belonging to the same mammalian-pathogenic *Picornaviridae* family. Three enzymes collectively convert tyrosine into betalain, a bright red pigment. Unlike traditional reporters such as *LacZ* or luciferase, which require external substrates, RUBY exploits endogenous tyrosine, enabling substrate-independent, equipment-free, and noninvasive monitoring of reporter activity without the need for UV illumination, imaging devices, or complex image analysis. This visible pigment accumulation allows for simplified experimental workflows through direct visual inspection. Recently, enhanced RUBY (eRUBY) rice and maize lines were reported with betalain-enriched endosperm by co-expressing a feedback-insensitive *TyrA* arogenate dehydrogenase from red beet (*BvADHα*) to boost tyrosine availability. These versions still employed a combination of P2A and F2A sequences to reduce construct instability caused by repetitive elements for 4 gene expression (Tan et al., 2025, Xue et al., 2026). Previously, our group identified a highly efficient 2A sequence derived from the Lepidopteran-infecting, non-mammalian-pathogenic *Thosea asigna* virus, referred to as T2A (Jeong et al., 2021). To address biosafety concerns in crop applications, we employed T2A in place of P2A or F2A for polycistronic expression of the three betalain biosynthetic genes, hereafter referred to as tRUBY (Fig. 1A and B).

Building on the refined tRUBY framework, we established *in planta* platforms for analyzing PDIs and PPIs in *Nicotiana benthamiana*, offering a functional alternative to yeast-based assays. tRUBY-1H monitors transcriptional regulation via PDIs (Fig. 1C), while tRUBY-2H detects protein complex formation through for PPIs (Fig. 1D). Both systems drive betalain accumulation, enabling direct visual and quantitative readouts in agroinfiltrated leaves (Fig. 1C and D). Together, these tools provide a versatile, low-cost, and non-destructive *in planta* system for robust detection of PDIs and PPIs under conditions that closely reflect the native plant cellular environment. We also present an optimized *Agrobacterium*-mediated transient expression protocol, validated in *N benthamiana*, as a broadly applicable resource for gene function and interactions studies with minimal equipment.

## MATERIALS AND METHODS

### Plant Material

To facilitate the effective application of the tRUBY-1H and tRUBY-2H systems, we describe a streamlined protocol for *Agrobacterium*-mediated expression in *N benthamiana* (strain LAB). This workflow encompasses plant preparation, *Agrobacterium* infiltration, tRUBY induction, and quantitative analysis of betalain accumulation (Fig. 1G). Healthy plant material is essential for the success of agroinfiltration-based assays. *Nicotiana benthamiana* seeds are sown on well-watered potting mix and germinated in a controlled growth chamber at 25°C under a 16 hours light/8 hours dark photoperiod. After 1 week, seedlings are transplanted into individual pots and cultivated for approximately 4 weeks until the leaves reach a diameter of ∼8 cm, which is optimal for infiltration. To maintain leaf pliability, ensuring efficient infiltration, plants should be protected from excessive light intensity that may induce leaf hardening.

### Agroinfiltration

For rapid assay implementation, a simplified *Agrobacterium* preparation method is employed. *Agrobacterium tumefaciens* strain GV3101 harboring the appropriate constructs is grown on agar plates containing the required antibiotics at 26°C until sufficient colony growth is achieved. Bacterial cells are then collected directly from the plates and resuspended in infiltration buffer (10 mM MES, pH 5.7; 10 mM MgCl_2_; 150 µM acetosyringone; 0.7 mM L-ascorbic acid), thereby eliminating the need for liquid culture. The suspension is incubated in the dark at room temperature for 2-3 hours and subsequently adjusted to a final OD_600_ of 0.5. For infiltration, bacterial suspensions are mixed at defined ratios depending on the assay: 1:1:2 (Reporter:Effector:P19) for the tRUBY-1H system and 1:0.5:0.5:2 (Reporter:Prey:Bait:P19) for the tRUBY-2H system. The mixtures are infiltrated into the abaxial surface of *N benthamiana* leaves using a needleless syringe, as previously described (Qu and Morris, 2002).

### RUBY Induction

Following infiltration, plants are incubated in the dark for 24 hours to promote efficient T-DNA transfer. Subsequently, plants are maintained under standard growth conditions (23 ± 2°C; 16-hour light/8-hour dark photoperiod) for 48-72 hours to allow reporter gene expression. Betalain pigment accumulation typically becomes visible within 3 days post-infiltration, enabling qualitative assessment by the naked eye.

### Betalain Quantification

For quantitative analysis, leaf disks are harvested, ground, and extracted in 40% (v/v) methanol, followed by incubation in the dark at 4°C for a minimum of 24 hours to ensure complete pigment extraction. The resulting supernatant is then transferred to a 96-well microplate, and absorbance is measured at 538 nm using a plate reader. Betalain content (mg/kg fresh tissue) is calculated based on the extinction coefficient of betanin (ε = 60,000 M^−1^cm^−1^), with the path length adjusted according to the sample volume (eg, 0.5286 cm for 200 µL), following the method of Pramanik et al. (2024).

$$\mathrm{Betalain}\;\mathrm{Contents}\;\left(\mathrm{mg}/\mathrm{kg}\right)=\frac{A_{538}\times \mathrm{Dilution}\;\mathrm{Factor}\times 550\times 1000}{60000\times 0.5286}$$

## RESULT

To facilitate the analysis of diverse molecular interactions, we developed 2 distinct vector systems compatible with the optimized workflow (Fig. 1E and F). The tRUBY-1H system is designed to visualize PDIs monitoring the transcriptional activity of promoters regulated by transcription factors. Its reporter vector (*pSH1*) contains a multiple cloning site upstream of the tRUBY reporter gene, enabling facile insertion of a target promoter. The cognate effector vectors (*pSH2-4*) express transcription factor under the constitutive CaMV 35S promoter, each fused to a C-terminal epitope tag (3xHA, 6xMyc, or GST) for immunodetection (Fig. 1E). Binding of the transcription factor to the target promoter activates tRUBY expression, resulting in betalain biosynthesis (Fig. 1C). For PPIs, we constructed the tRUBY-2H system based on the GAL4/UAS architecture (Fig. 1F). The universal reporter vector (*pSH5*) carries 4 copies of the upstream activating sequence (4xUAS) fused to a minimal 35S promoter upstream of the tRUBY gene. Interaction between a prey protein fused to the VP16 activation domain (AD) and a bait protein fused to the GAL4 DNA-binding domain (BD) recruits the AD to the UAS region, thereby inducing tRUBY expression (Fig. 1D). A series of prey vectors (*pSH6-8*) and bait vectors (*pSH9-11*) with different epitope tags (3xHA, 6xMyc, or GST) were generated to provide experimental flexibility (Fig. 1F).

**Fig. 2:**
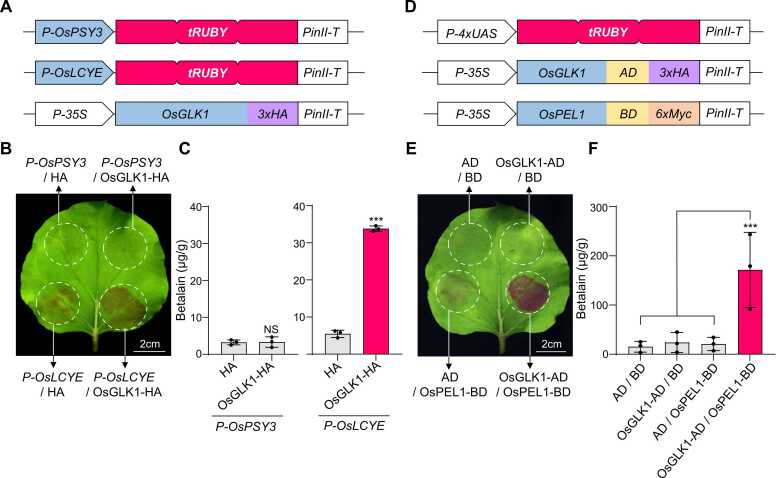
*Validation of the tRUBY-1H and tRUBY-2H systems using established molecular interactions.* (A-C) Validation of the tRUBY-1H system using the transcription factor OsGLK1 and its target promoters. (A) Schematic of the constructs used. The *OsPSY3* and *OsLCYE* promoters were cloned upstream of tRUBY, and *OsGLK1* was expressed as the effector. (B) Representative image of an *N benthamiana* leaf. Red pigmentation is exclusively observed only in the sector co-expressing OsGLK1 and the *OsLCYE* promoter-driven reporter. Scale bar = 2 cm. (C) Quantification of betalain accumulation. Betalain levels were measured in leaf disks expressing *P-OsPSY3* (left) or *P-OsLCYE* (right) in the presence of OsGLK1-HA or the HA control. Data represent means ± SD (*n* = 3). Asterisks indicate a significant difference compared to the control (***P < .001; NS, not significant; Student’s *t*-test). (D-F) Validation of the tRUBY-2H system using the OsGLK1-OsPEL1 interaction. (D) Schematic of the tRUBY-2H constructs. OsGLK1 and OsPEL1 were fused to the activation domain (AD) and DNA-binding domain (BD), respectively. (E) Representative image of the tRUBY-2H assay. Intense red pigmentation indicates the specific interaction between GLK1-AD and PEL1-BD. Scale bar = 2 cm. (F) Quantification of betalain levels. Co-expression of the interacting pair (GLK1-AD/PEL1-BD) resulted in robust betalain production compared to negative controls. Data represent means ± SD (*n* = 3, ***P < .001).

Given its pleiotropic roles in crop improvement and involvement in complex PDIs and PPIs, the transcription factor *Oryza sativa* GOLDEN2-LIKE1 (OsGLK1) was selected as a model for testing the tRUBY-1H system (Choi et al., 2024, Kim et al., 2023). Promoter targets were chosen based on prior ChIP-seq data (Tu et al., 2022), which revealed weak or negligible OsGLK1 binding peaks in the *OsPSY3* promoter but strong peaks in the *OsLCYE* promoter. To experimentally confirm these binding profiles, tRUBY-1H reporter constructs were generated using the promoters of *OsPSY3* and *OsLCYE* to evaluate OsGLK1-mediated transcriptional regulation of carotenoid biosynthetic genes (Fig. 2A). Transient co-expression assays showed that the *OsLCYE* promoter, when co-expressed with OsGLK1, induced distinct red pigmentation, whereas the *OsPSY3* promoter did not result in any visible pigment accumulation (Fig. 2B). Consistently, betalain quantification revealed a significant increase in pigment content for the *OsLCYE* promoter (∼35 µg/g fresh weight, *P* < .001), while the *OsPSY3* promoter exhibited no significant difference compared to the control (Fig. 2C). This clear phenotypic and quantitative contrast highlights the specificity of tRUBY-1H system in distinguishing between low- and high-affinity transcriptional targets, consistent with the ChIP-Seq data.

To assess the utility of the tRUBY-2H system in detecting PPIs, we selected OsGLK1 a multifunctional transcription factor involved in diverse regulatory networks (Kim et al., 2023) and its previously characterized interacting partner, *Oryza sativa* PSEUDO-ETIOLATION IN LIGHT 1 (OsPEL1). This interaction was previously confirmed through multiple complementary assays, including yeast 2H, BiFC, and split-luciferase assays (Choi et al., 2025). Prey and bait vectors were constructed to express OsGLK1 fused to the VP16-AD and OsPEL1 fused to the GAL4-DNA-BD, along with a UAS-driven tRUBY reporter (Fig. 2D). Co-expression of these constructs in *N benthamiana* leaves resulted in intense red pigmentation, whereas negative controls—including empty vectors or single constructs exhibited little to no visible color change (Fig. 2E). Quantitative analysis confirmed that the OsGLK1-OsPEL1 interaction induced robust betalain production (>170 µg/g fresh weight, *P* < .001), significantly exceeding background levels observed in all control groups (<20 µg/g fresh weight) (Fig. 2F). This high signal-to-noise ratio, consistent with results from conventional PPI assays, demonstrates the sensitivity and specificity of the tRUBY-2H system.

## DISCUSSION

We present the design and application of the tRUBY-1H and tRUBY-2H systems for the visual monitoring of PDIs and PPIs in *N benthamiana*. While the RUBY reporter has recently been utilized to analyze molecular interactions (Chen et al., 2023, Liu et al., 2024), our tRUBY-1H and tRUBY-2H systems provide a standardized vector toolkit to readily facilitate these assays. Incorporating the non-pathogenic T2A sequences into the reporter architecture enhanced biosafety compared to previous F2A or P2A-based constructs. A key advantage of the tRUBY platform over conventional reporters such as BiFC or split-luciferase is its non-destructive, instrument-free readout. Betalain pigment accumulation allows intuitive detection of positive interactions by the naked eye, facilitating high-throughput screening of large candidate libraries without the need for cell lysis or specialized fluorescence imaging equipment. While betalain-based detection offers many advantages, it lacks subcellular resolution, as metabolite-based reporter, which pigments primarily accumulate in the vacuole. Furthermore, signal intensity may vary depending on tyrosine availability and host physiology, requiring controlled growth conditions for reproducibility. Additionally, because absorbance at 538 nm can be influenced by endogenous anthocyanins and pH variations, employing strict negative controls for background subtraction and maintaining consistent extraction conditions are highly recommended for accurate quantification. Despite these limitations, the tRUBY system reporter opens new opportunities for further downstream analyses: interaction-positive tissues remain viable and can be harvested for immunoblotting or co-immunoprecipitation, aided by integrated epitope tags (eg, HA, Myc, GST). Collectively, tRUBY-1H and tRUBY-2H systems provide a versatile *in planta* platform that bridges rapid visual screening with molecular-level validation, enabling both high-throughput and mechanistic studies for plant biology. Furthermore, the visually scorable nature of the tRUBY system extends its utility beyond transient assays, presenting a valuable tool for future applications such as enhancer trap screening and the generation of variegated transgenic plants.

## Funding and support

This work was supported by the National Research Foundation of Korea (NRF) grant funded by the Korea government (MSIT) (RS-2024-00347806, RS-2024-00440478, and RS-2024-00407469 to S.-H.H.; RS-2023-00212636 to H.C.).

## CRediT authorship contribution statement

**Sangyun Kim:** Writing – review & editing, Writing – original draft, Methodology, Investigation, Formal analysis, Data curation, Conceptualization. **Heebak Choi:** Writing – review & editing, Writing – original draft, Funding acquisition, Conceptualization. **Tae Gyu Yi:** Visualization, Investigation. **Sun-Hwa Ha:** Writing – review & editing, Supervision, Project administration, Funding acquisition, Conceptualization.

## Declaration of Competing Interests

The authors declare that they have no known competing financial interests or personal relationships that could have appeared to influence the work reported in this paper.
